# Knowledge and Attitudes towards Handling Eggs in the Home: An Unexplored Food Safety Issue?

**DOI:** 10.3390/ijerph14010048

**Published:** 2017-01-06

**Authors:** Harriet Whiley, Beverley Clarke, Kirstin Ross

**Affiliations:** 1 Environmental Health, School of the Environment, Flinders University, Adelaide 5001, Australia; Kirstin.Ross@flinders.edu.au; 2 Geography, School of the Environment, Flinders University, Adelaide 5001, Australia; Beverley.Clarke@flinders.edu.au

**Keywords:** food safety, salmonella, eggs, foodborne illness, consumer

## Abstract

Foodborne illness is a global public health issue, with food handling in the home identified as an underestimated source. In Australia, there has been a significant increase in the incidence of salmonellosis with the majority of outbreaks linked to eggs. This study investigated Australian eggs consumer attitudes, behaviours and risk perceptions associated with the handling of raw eggs in the home. It was identified that 67% of participants chose free range eggs, 11% kept poultry, 7% did not have any preference, 7% cage eggs, 4% barn eggs, 2% organic eggs and 1% pasteurized eggs. The majority of participants (91%) reported they stored eggs in the fridge. It was identified that there is an underestimation of “risky behaviour” associated with the consumption of raw eggs in the home, as 84% of participants indicated that they did not consume raw eggs, but subsequently 86% indicated that they had eaten mixture/batter containing raw eggs. Participants’ responses relating to food safety were also examined in relation to their profession and gender. Safer food handling practices were identified by Environmental Health Officers (EHO) and Food handlers compared to all other professions (*p* < 0.05). However, the gender of participants did not significantly affect (*p* > 0.05) their responses.

## 1. Introduction

Worldwide, foodborne illness is a significant public health burden [1]. Global estimates indicating that every year there are just under 600 million cases, including 350,000 fatalities [2]. In Australia it was estimated that annually there are 4.1 million cases of foodborne illness including 30,000 hospitalizations and 100 fatalities [3]. One of the most prevalent causes of foodborne illness is salmonellosis [4,5]. Over the last decade in Australia the incidence of salmonellosis has increased from 40.9 per 100,000 population in 2005 to 71.5 per 100,000 population in 2015 [6]. One of the most common sources of salmonellosis has been identified as raw eggs and egg products [7,8]. Moffatt et al. [9] demonstrated that between 2001 and 2011 there was a significant increase in the proportion of foodborne *Salmonella* outbreaks linked to eggs (*p* < 0.001), with up to 45% of foodborne salmonellosis outbreaks being linked to eggs by 2011. Additionally, in 2011 the OzFoodNet working group demonstrated that 48% of all salmonellosis outbreaks could be attributed to eggs or egg related products [8]. Current studies investigating mechanisms to prevent *Salmonella* contamination during the egg production process are somewhat contradictory [10,11]. This demonstrates the importance of appropriate food handling practices and post egg collection control methods [11]. 

Globally, the primary causative agent for salmonellosis is *Salmonella enterica* serovar Enteritidis [7]; however, in Australia it is *Salmonella enterica* serovar Typhimurium [9]. Experimental studies have demonstrated that both serovars have the potential to internally contaminate eggs, but predominately *S.* Enteritidis is found internally within the egg [7] and *S.* Typhimurium is found on the outside of the egg shell through cross contamination [10]. There is also the potential for *S.* Typhimurium to penetrate the eggshell resulting in internal contamination [12]. The *Salmonella* serovar influences the effectiveness of control methods as each behaves differently and presents different challenges to the production process [11,13,14].

There have been numerous studies which have demonstrated that a significant proportion of foodborne illness can be attributed to improper food safety practices in the home [15]. In the European Union more than half of all *Salmonella* outbreaks are linked to contamination in the home [16]. In the USA from 1998–2008, 15% of reported foodborne illness outbreaks were from foods prepared in the home, with *Salmonella* identified as one of the main causative agents [17]. In Australia the percentage was even higher, with 28% of the egg associated salmonellosis outbreaks from 2001–2011 linked to private residences [9]. In fact, the true incidence of foodborne illness originating from the home may be much higher than reported due to many cases going undiagnosed [16]. 

There are numerous factors which may contribute to the home being a significant source of foodborne illness [16,18,19]. The majority of food consumed by people is prepared at home. There is therefore an increased opportunity for food preparation and handling safety mistakes to result in illness [16]. This is compounded by people not considering their food preparation practices as risky, or, not considering themselves or their family at risk from foodborne illness [20]. Previous studies have investigated consumer attitudes towards the handling of high risk foods in the home [18,21]. However, there is a limited number of studies that specifically investigate consumer attitudes toward handling eggs in the home, and even fewer studies specifically from an Australian perspective. This paper provides insights from a survey investigating people’s egg handling and cooking practices when using raw eggs in food preparation in the home, and their knowledge and attitudes regarding the associated risks. Information gained form this study will help inform future efforts to reduce the incidence of salmonellosis in Australia.

## 2. Materials and Methods 

### 2.1. Design

Egg consumer food safety knowledge, perceptions and food handling behaviours with regards to use of raw eggs in food preparation in the home were assessed using an online survey. The survey was approved by the Flinders University Social and Behavioural Research Ethics Committee (SBREC) (project number: 7254) on 1 June 2016 and subsequently made available from 15 July to 31 October 2016. The survey was promoted on a number of Facebook pages: including: Environmental Health Australia, The Adelaide Showground Farmers Market and community Facebook pages. This resulted in a snowball sample, where individuals shared the survey links with their friends and so on. The survey questions are shown in Supplementary Materials.

### 2.2. Statistical Analysis

To determine statistical significance, cumulative responses for each question were graphed on Microsoft Excel until a line of apparent linearity was observed. Pearson’s chi-square analysis was also conducted using Microsoft Excel to compare participant responses to different questions depending on profession and gender. 

## 3. Results

### 3.1. Participants

A total of 294 people responded and 282 completed the survey. Participants were required to confirm they were 18 years of age or older. Participants’ demographics are shown in Table 1. There was an even distribution of age groups; however, 78% were female. Two-thirds of survey participants had a tertiary qualification. 

To ensure adequate numbers of people were surveyed, cumulative values were graphed until the resultant curve appeared linear. This line of apparent linearity was found for all measures except for questions 5 and 6 which specifically related to keeping poultry and were only available to a subset of participants who indicated that they kept their own chickens. For all other questions, given the line of apparent linearity, increasing the number of survey participants would be unlikely to change the outcome.

### 3.2. Egg Consumer Preferences

The survey results indicate that the majority of consumers have a preference for free range eggs with 68.6% (194/282) of survey participants indicating that they predominately buy free range eggs. The remainder, either keep poultry (30/282; 10.7%), do not have any preference about the eggs they buy or use (20/282; 7.1%), buy cage eggs (19/282; 6.8%), barn eggs (11/282; 4.0%), organic eggs (6/282; 2.1%) or pasteurised eggs (2/282; 0.7%). The overwhelming majority of participants (91%) reported they stored their eggs in the fridge.

To be statistically sound more responses were needed for the results of the two subset questions relating to keeping poultry. However, answers showed that 50% of participants were currently using unsafe practises with regards to handling dirty eggs. Five participants (from a total of 30 who kept poultry) indicated that if an egg was dirty they would use it without washing it first while nine said they would wash a dirty egg. Of the remaining 50% of survey participants who reported safe egg handling practises, 13 would wipe a dirty egg, and one would discard it. The responses regarding the use of cracked eggs indicated safer practise with only three participants stating they would use an egg even if it was cracked. Three participants indicated they would feed a cracked egg to a pet, and the remaining 23 participants said they would discard a cracked egg. This information demonstrates that there are some poultry keepers who are not aware of all the risks associated with egg handling.

### 3.3. Food Safety Knowledge

**Figure 1:**
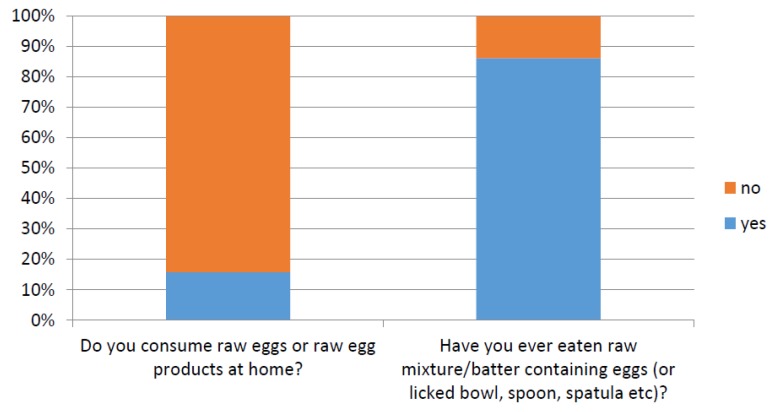
The percentages of participant responses for the questions “Do you consume raw eggs or raw egg products at home?” and “Have you ever eaten raw mixture/batter containing eggs (or licked bowl, spoon, spatula, etc.)?”

Several questions were designed to assess egg consumer knowledge and attitudes towards food safety and use of raw eggs in the home. One question asked “Do you consume raw eggs or raw egg products in the home?” with 84% of participants responding “no”. However, another question asked “Have you ever eaten raw mixture/batter containing eggs (or licked bowl, spoon, spatula, etc.)?”, and 86% of participants responded “yes” (as shown in Figure 1). This demonstrates that many egg consumers do not know, or acknowledge, they are undertaking potentially “risky” behaviours (such as consuming raw eggs) in the home.

**Figure 2:**
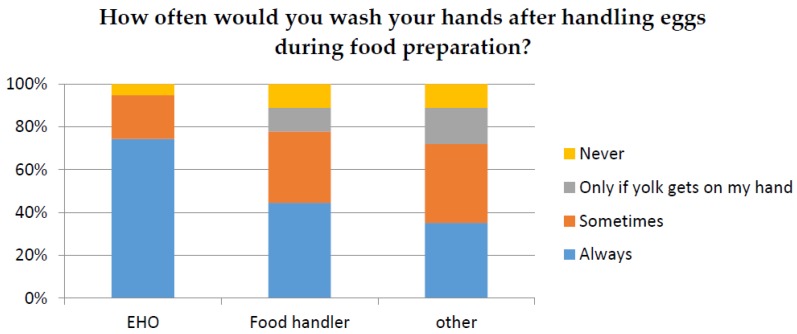
Demonstrates the percentage of responses for the question “How often would you wash your hands after handling eggs during food preparation?” for respondent who identified their profession as an “Environmental Health Officer (EHO)”, Food handler and all other professions.

**Figure 3:**
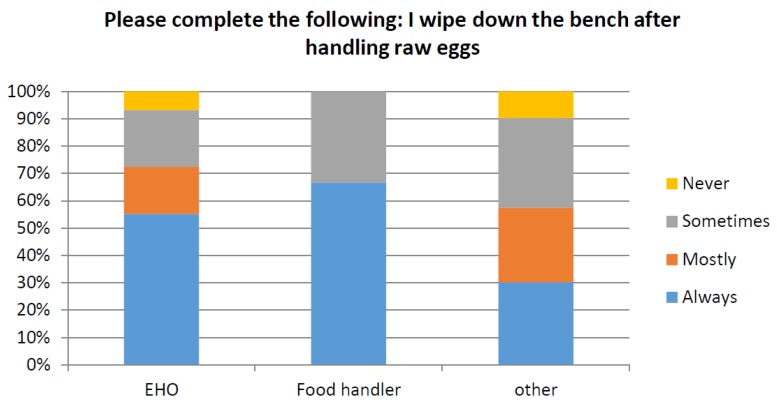
Demonstrates the percentage of responses for the question “Please complete the following: I wipe down the bench after handling raw eggs?” for respondent who identified their profession as an “Environmental Health Officer (EHO)”, Food handler and all other professions.

**Figure 4:**
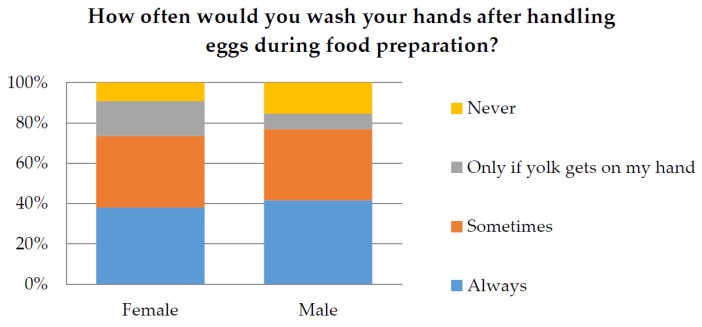
Demonstrates the percentage of male and female responses for the question “How often would you wash your hands after handling eggs during food preparation?”

**Figure 5:**
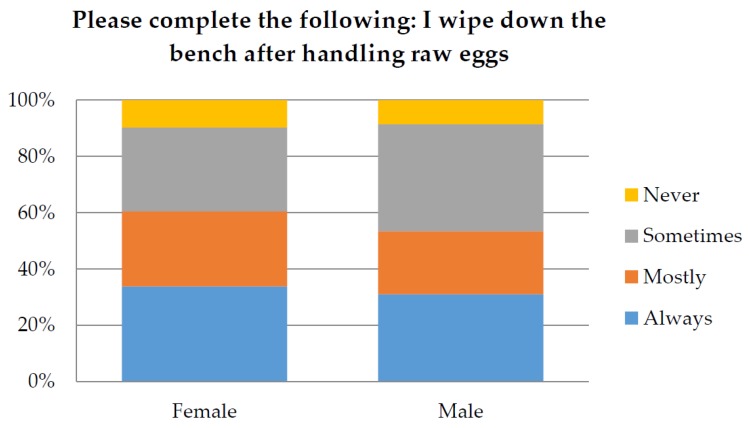
Demonstrates the percentage of male and female responses for the question “Please complete the following: I wipe down the bench after handling raw eggs?”

The responses relating to food safety were also examined in relation to participant’s profession and gender. Responses from participants who identified as Environmental Health Officers (EHO) or Food handler were compared to all other professions. For the question “How often would you wash your hands after handling eggs” only 38.7% of total participants responded “Always”. The chi-squared analysis demonstrated that there was a significant difference (*p* < 0.05) between responses according to profession (Figure 2), with EHO’s responding “Always” being the most significant factor. Only 34% of participants responded that they always “wipe down the bench after handling raw eggs”. The chi-squared analysis also demonstrated a significant difference (*p* < 0.05) between responses according to different professions (Figure 3). However, gender did not make a significant difference to the results (*p* > 0.05) as shown in Figure 4 and Figure 5.

## 4. Discussion

The findings from this Australian survey show that respondents have a preference for free range eggs and that 91% store their eggs in the refrigerator. This is supported by a study by Lu [22] that demonstrated that Canadian consumers were willing to pay a premium for free range eggs. Another study conducted in Finland also reported that 93% of consumers stored their eggs at chilled temperatures [23]. However, the Australia New Zealand Food Standards Code (Standard 2.2.2—Eggs and egg products) states that although it is recommended that eggs are stored chilled, there is no legislative requirement as Australian intact eggs are unlikely to be internally infected with *Salmonella* [24]. These guidelines do not take into consideration the potential for *S.* Typhimurium to penetrate through the eggshell resulting in internal contamination [12,25].

In the USA it has been identified that there is an increasing interest in domestic “backyard” poultry [26]. In this study 10.7% of participants indicated they kept laying poultry. From the survey responses it is clear there is a lack of knowledge surrounding the risks of handling and using eggs collected from domestic “backyard” poultry. Although there were not enough participants for these specific questions to provide statistically significant conclusions, this study identified that this is an area warranting further investigation. These findings support a study conducted in the USA that identified only 50% of participants were aware of the connection between poultry and human salmonellosis [27]. 

A previous study explored factors that influence an individual’s perception of “risky behaviour” with regards to natural hazards, with personal experience being identified as one of the most important factors [28]. However, this is the first study to explore Australian individuals’ perception of risk with regards to handling raw eggs. This study observed that respondents were potentially not recognizing or not acknowledging risks associated with handling eggs and cooking with raw egg products in the home. This was demonstrated by 84% of participants claiming to not consume raw egg products in the home but 86% who contradicted this claim by affirming they lick bowls or utensils containing raw egg mixture. A potential limitation of this interpretation is an individual’s understanding of questions in regard to the implied frequency of behaviours. For example, some questions placed no time limit or constraints on the occurrence of a type of behaviour: “Do you consume raw eggs or raw egg products at home?” (This could be interpreted to imply regular occurrence). This is in contrast to the “ever” in, “Have you ever eaten raw mixture/batter containing eggs (or licked bowl, spoon, spatula, etc.)?” However, the observation that respondents were potentially not recognizing risky food handling behaviours could be explained by the fact that many cases of foodborne illness do not get reported and as such, the source of infection is not always identified. This would influence personal experiences and hence attitudes towards food safety [29]. 

With regard to hand washing or bench wiping, there was no significant difference between the food safety knowledge of male or female participants. This difference is particularly interesting for the question relating to the washing of hands after handling raw eggs as several previous studies point to gender differences in hand washing behaviour [30,31,32,33]. Studies from New Zealand [30] and Korea [32] demonstrate that females were more likely to wash their hands after using public restrooms compared to males. Another study from the USA demonstrated that female hospital staff in a critical care unit were more likely to wash their hands after patient contact compared to male staff [33]. Additionally, a study conducted in the USA of young adults (mean age 19.9) regarding their food safety behaviours found that females significantly identified safer food handling practices compared to males [18]. The lack of gender difference in this study may be due to the sampling method (this was a self-reporting survey). Additionally the survey findings would have been influenced by a recruitment bias which resulted in a disproportionate percentage of EHO participates who would have different perspective and understanding of food safety risks compared to the “average household consumer”.

EHOs and Food handlers identified significantly safer food handling practices compared to other professions. This demonstrates that food safety education is essential for improving food safety in the home. Education campaigns regarding the risk of salmonellosis from eggs will need to be tailored specifically for Australia, as worldwide, the most prevalent cause of salmonellosis is *S.* Enteritidis; but, in Australia it is *S.* Typhimurium [11]. These serovars behave differently and as such different control mechanisms are required [34]. 

## 5. Conclusions

In Australia the incidence of salmonellosis is increasing with the majority of outbreaks linked to eggs. The findings from this study suggest that Australian consumers may not be utilizing safe food handling practices when handling raw eggs in the home. This may be contributing to the burden of salmonellosis in Australia. There was no significant difference observed between the food safety practices of male compared to female participants. The recruitment bias of the survey resulted in a disproportionate percentage of EHO participants. Significant differences were observed for EHO and Food handler responses compared to other professions. This demonstrates that professions receiving food safety education utilize safer food handling practices in the home.

## Figures and Tables

**Table 1:** Demographics of survey participants (n = 282).

Characteristics		No. of Participants (%)
Gender	Male	65 (23.0%)
	Female	216 (76.6%)
	Other	1 (0.4%)
Age	25 or under	48 (17%)
	26–35	67 (23.8%)
	36–45	55 (19.5%)
	46–55	78 (27.7%)
	56–65	30 (10.6%)
	Over 65	4 (1.4%)
Highest level of education completed	Less than Year 12 or equivalent	18 (6.4%)
	Year 12 or equivalent	35 (12.4%)
	Vocational Qualification	41 (14.5%)
	Undergraduate Diploma	140 (49.6%)
	Master’s Degree	41 (14.5%)
	Doctorate	7 (2.5%) *
Employment	Environmental Health Officer (EHO)	29 (10.3%)
	Food Handler	9 (3.2%)
	Other	244 (86.5%)

* Due to rounding total equals 99.9%.

## Supplementary Materials

The following are available online at www.mdpi.com/1660-4601/14/1/48/s1.

## References

[1] Havelaar A.H., Kirk M.D., Torgerson P.R., Gibb H.J., Hald T., Lake R.J., Praet N., Bellinger D.C., de Silva N.R., Gargouri N. (2015). World Health Organization global estimates and regional comparisons of the burden of foodborne disease in 2010. PLoS Med..

[2] Kirk M.D., Pires S.M., Black R.E., Caipo M., Crump J.A., Devleesschauwer B., Döpfer D., Fazil A., Fischer-Walker C.L., Hald T. (2015). World Health Organization estimates of the global and regional disease burden of 22 foodborne bacterial, protozoal, and viral diseases, 2010: A data synthesis. PLoS Med..

[3] Kirk M., Ford L., Glass K., Hall G. (2014). Foodborne illness, Australia, circa 2000 and circa 2010. Emerg. Infect. Dis..

[4] Galiş A.M., Marcq C., Marlier D., Portetelle D., Van I., Beckers Y., Théwis A. (2013). Control of *Salmonella* contamination of shell eggs—Preharvest and postharvest methods: A review. Compr. Rev. Food Sci. Food Saf..

[5] Majowicz S.E., Musto J., Scallan E., Angulo F.J., Kirk M., O’Brien S.J., Jones T.F., Fazil A., Hoekstra R.M. (2010). The global burden of nontyphoidal *Salmonella* gastroenteritis. Clin. Infect. Dis..

[6] Department of Health, Australia’s National Notifiable Diseases Surveillance System (NNDSS) National Notifiable Diseases Surveillance System Data. http://www9.health.gov.au/cda/source/cda-index.cfm.

[7] Howard Z.R., O’Bryan C.A., Crandall P.G., Ricke S.C. (2012). *Salmonella* Enteritidis in shell eggs: Current issues and prospects for control. Food Res. Int..

[8] OzFoodNet Working Group (2015). Monitoring the incidence and causes of diseases potentially transmitted by food in Australia: Annual report of the ozfoodnet network, 2011. Commun. Dis. Intell. Q. Rep..

[9] Moffatt C.R., Musto J., Pingault N., Miller M., Stafford R., Gregory J., Polkinghorne B.G., Kirk M.D. (2016). *Salmonella* Typhimurium and outbreaks of egg-associated disease in Australia, 2001 to 2011. Foodborne Pathog. Dis..

[10] Chousalkar K.K., Sexton M., McWhorter A., Hewson K., Martin G., Shadbolt C., Goldsmith P. (2015). *Salmonella* Typhimurium in the Australian egg industry: Multidisciplinary approach to addressing the public health challenge and future directions. Crit. Rev. Food Sci. Nutr..

[11] Whiley H., Ross K. (2015). *Salmonella* and eggs: From production to plate. Int. J. Environ. Res. Public Health.

[12] Whiley A., Fallowfield H., Ross K., McEvoy V., Whiley H. (2016). Higher storage temperature causes greater *Salmonella enterica* serovar Typhimurium internal penetration of artificially contaminated, commercially available, washed free range eggs. J. Food Prot..

[13] Okamura M., Kamijima Y., Miyamoto T., Tani H., Sasai K., Baba E. (2001). Differences among six *Salmonella* serovars in abilities to colonize reproductive organs and to contaminate eggs in laying hens. Avian Dis..

[14] Carrique-Mas J., Breslin M., Snow L., McLaren I., Sayers A., Davies R. (2009). Persistence and clearance of different *Salmonella* serovars in buildings housing laying hens. Epidemiol. Infect..

[15] Redmond E.C., Griffith C.J. (2003). Consumer food handling in the home: A review of food safety studies. J. Food Prot..

[16] Byrd-Bredbenner C., Berning J., Martin-Biggers J., Quick V. (2013). Food safety in home kitchens: A synthesis of the literature. Int. J. Environ. Res. Public Health.

[17] Gould L.H., Walsh K.A., Vieira A.R., Herman K., Williams I.T., Hall A.J., Cole D. (2013). Surveillance for foodborne disease outbreaks—United States, 1998–2008. Morb. Mortal. Wkly. Rep..

[18] Byrd-Bredbenner C., Maurer J., Wheatley V., Schaffner D., Bruhn C., Blalock L. (2007). Food safety self-reported behaviors and cognitions of young adults: Results of a national study. J. Food Prot..

[19] Brewer M.S., Rojas M. (2008). Consumer attitudes toward issues in food safety. J. Food Saf..

[20] De Jong A., Verhoeff-Bakkenes L., Nauta M., De Jonge R. (2008). Cross-contamination in the kitchen: Effect of hygiene measures. J. Appl. Microbiol..

[21] Klontz K.C., Timbo B., Fein S., Levy A. (1995). Prevalence of selected food consumption and preparation behaviors associated with increased risks of food-borne disease. J. Food Prot..

[22] Lu Y. (2013). Consumer Preference for Eggs from Enhanced Animal Welfare Production System: A Stated Choice Analysis. Masters’ Thesis.

[23] Lievonen S., Havulinna A., Maijala R. (2004). Egg consumption patterns and salmonella risk in Finland. J. Food Prot..

[24] Food Standards Australia New Zealand (FSANZ) (2016). Standard 2.2.2—Eggs and egg products. Food Standards Code.

[25] Gole V.C., Chousalkar K.K., Roberts J.R., Sexton M., May D., Tan J., Kiermeier A. (2014). Effect of egg washing and correlation between eggshell characteristics and egg penetration by various *Salmonella* Typhimurium strains. PLoS ONE.

[26] Pollock S., Stephen C., Skuridina N., Kosatsky T. (2012). Raising chickens in city backyards: The public health role. J. Community Health.

[27] Beam A., Garber L., Sakugawa J., Kopral C. (2013). *Salmonella* awareness and related management practices in U.S. Urban backyard chicken flocks. Prev. Vet. Med..

[28] Wachinger G., Renn O., Begg C., Kuhlicke C. (2013). The risk perception paradox—Implications for governance and communication of natural hazards. Risk Anal..

[29] Redmond E.C., Griffith C.J. (2004). Consumer attitudes and perceptions towards microbial food safety in the domestic kitchen. J. Food Saf..

[30] Garbutt C., Simmons G., Patrick D., Miller T. (2007). The public hand hygiene practices of New Zealanders: A national survey. N. Z. Med. J..

[31] Van de Mortel T., Bourke R., McLoughlin J., Nonu M., Reis M. (2001). Gender influences handwashing rates in the critical care unit. Am. J. Infect. Control.

[32] Jeong J.S., Choi J.K., Jeong I.S., Paek K.R., In H.K., Park K.D. (2007). A nationwide survey on the hand washing behavior and awareness. J. Prev. Med. Public Health.

[33] Altekruse S.F., Street D.A., Fein S.B., Levy A.S. (1996). Consumer knowledge of foodborne microbial hazards and food-handling practices. J. Food Prot..

[34] Martelli F., Davies R.H. (2012). *Salmonella* serovars isolated from table eggs: An overview. Food Res. Int..

