# Supplementary material for: Knowledge and Attitudes towards Handling Eggs in the Home: An Unexplored Food Safety Issue?
Source: Int J Environ Res Public Health. 2017 Jan 6;14(1):48. doi: 10.3390/ijerph14010048 (PMC5295299; doi:10.3390/ijerph14010048)
Supplement: Supplementary file 1 [file ijerph-14-00048-s001.pdf]

Supplementary Materials: Knowledge and Attitudes towards Handling Eggs in the Home: An Unexplored Food Safety Issue?

Harriet Whiley, Beverley Clarke and Kirstin Ross

Survey Questions: Consumer's Attitudes towards Eggs and Food Safety in the Home

Hi,

We are a team of researchers from Flinders University conducting a survey on consumer's attitudes towards eggs and food safety in the home. Are you over 18 and available to spare a couple of minutes of your time to complete this short survey?

Thank you very much for your time.

Dr. Harriet Whiley, Dr. Kirstin Ross and Associate Professor Beverley Clarke

The following questions relate to your choices when purchasing eggs

1. What type of eggs do you mostly buy?
   Organic (go to question 4)
   Free range (go to question 4)
   Barn (go to question 4)
   Caged (go to question 4)
   Pasteurised (go to question 4)
   Any, I don't have a preference (go to question 4)
   I keep poultry and collect my own eggs (go to question 2)
2. If an egg is dirty, typically what do you do with it?
   Use it
   Discard it
   Wash it
   Wipe it
   Other____________ (please specify)
3. If an egg is cracked what do you do with it?
   Use it
   Discard it
   Wash it
   Wipe it
   Other____________ (please specify)
4. Where do you typically store your eggs?
   Fridge
   Shelf

The following questions relate to food preparation in the home

5. Do you consume raw eggs or raw egg products at home?
   Yes
   No
   Other__________________________________ (please specify)
6. How often would you wash your hands after handling eggs during food preparation?
   Never
   Sometimes

Always
If yolk gets on my hand

7. Have you even eaten raw mixture/batter containing eggs (or licked bowl, spoon, spatula, etc.)?
Yes
No

8. Please complete the following
I wipe down the bench after handling raw eggs
Always
Mostly
Sometimes
Never

The following questions relate to you

9. Gender?
Male
Female
Transgender

10. Age?
25 or under
26-35
36-45
46-55
56-65
Over 65

11. Occupation?

12. What is your highest level of education completed?
Less than Year 12 or equivalent
Year 12 or equivalent (*HSC/Leaving cert)
Vocational Qualification
Undergraduate diploma
Bachelor degree (including honours)
Postgraduate diploma
Master's degree
Doctorate

13. What is your country of residence?
Australia
Other____________ (please specify)

© 2017 by the authors; licensee MDPI, Basel, Switzerland. This article is an open access article distributed under the terms and conditions of the Creative Commons by Attribution (CC-BY) license (http://creativecommons.org/licenses/by/4.0/)
